# Women’s circles as a culturally safe psychosocial intervention in Guatemalan indigenous communities: a community-led pilot randomised trial

**DOI:** 10.1186/s12905-019-0744-z

**Published:** 2019-04-03

**Authors:** Anne Marie Chomat, Aura Isabel Menchú, Neil Andersson, Manuel Ramirez-Zea, Duncan Pedersen, Alexandra Bleile, Paola Letona, Ricardo Araya

**Affiliations:** 1 Participatory Research at McGill (PRAM), Department of Family Medicine, McGill University, 5858 Chemin de la Côte-des-Neiges-3rd floor, Suite 300, Montréal, QC H3S 1Z1 Canada; 2 CIET International Guatemala, 5ª calle 14-35, apartamento 304, Edificio Las Tapias, zona 3, Quetzaltenango, Guatemala; 3 Centro de Investigación de Enfermedades Tropicales (CIET), Universidad Autónoma de Guerrero, Acapulco, Mexico; 4 Research Center for the Prevention of Chronic Diseases (CIIPEC), Institute of Nutrition of Central America and Panama (INCAP), Calzada Roosevelt 6-25 zona 11, Apartado Postal 1188, Guatemala City, Guatemala; 5 Department of Psychiatry and Division of Social and Transcultural Psychiatry, McGill University, Montréal, Canada; 6 Centre for Global Mental Health, King’s College London, De Crespigny Park, London, SE5 8AF UK

**Keywords:** Maternal mental health, Indigenous women, Guatemala, Participatory research, Women’s circles, Co-design, Cultural safety

## Abstract

**Background:**

Indigenous Maya women in Guatemala show some of the worst maternal health indicators worldwide. Our objective was to **t**est acceptability, feasibility and impact of a co-designed group psychosocial intervention (Women’s Circles) in a population with significant need but no access to mental health services.

**Methods:**

A parallel group pilot randomised study was undertaken in five rural Mam and three periurban K’iche’ communities. Participants included 84 women (12 per community, in seven of the communities) randomly allocated to intervention and 71 to control groups; all were pregnant and/or within 2 years postpartum. The intervention consisted of 10 sessions co-designed with and facilitated by 16 circle leaders. Main outcome measures were: maternal psychosocial distress (HSCL-25), wellbeing (MHC-SF), self-efficacy and engagement in early infant stimulation activities. In-depth interviews also assessed acceptability and feasibility.

**Results:**

The intervention proved feasible and well accepted by circle leaders and participating women. 1-month post-intervention, wellbeing scores (*p*-value 0.008) and self-care self-efficacy (0.049) scores were higher among intervention compared to control women. Those women who attended more sessions had higher wellbeing (0.007), self-care and infant-care self-efficacy (0.014 and 0.043, respectively), and early infant stimulation (0.019) scores.

**Conclusions:**

The pilot demonstrated acceptability, feasibility and potential efficacy to justify a future definitive randomised controlled trial. Co-designed women’s groups provide a safe space where indigenous women can collectively improve their functioning and wellbeing.

**Trial registration:**

ISRCTN13964819. Registered 26 June 2018, retrospectively registered.

**Electronic supplementary material:**

The online version of this article (10.1186/s12905-019-0744-z) contains supplementary material, which is available to authorized users.

## Background

Perinatal mental disorders – depression, anxiety and somatic disorders – can be detrimental to women’s health, pregnancy outcomes and infant neurological, cognitive, emotional, and social development [1–4]. Maternal mental health has been linked to reduced responsiveness in caregiving and higher rates of behavioral problems in children [5] and young adults [6]*.* Maternal anxiety – which indigenous women may be at increased risk for [7] – has been associated with preterm birth [8] and, in low-income settings, maternal depression has been associated with low birth weight, childhood stunting, higher rates of diarrheal diseases and poor cognitive development in young children [1, 9].

A systematic review reported perinatal mental disorders were common in low- and lower-middle-income countries (LMIC), affecting 16% of pregnant women and 20% of women in the postpartum period [10]. Indigenous women experience higher rates of partner abuse than non-indigenous women, for whom partner violence is an especially strong predictor of poor mental health [11]. Protective factors include relative social and economic advantage, formal education, secure employment, reproductive health services, belonging to the ethnic majority, and having a respectful, trustworthy intimate partner [10]. Women may also be better able to counter stress if they have high self-esteem and self-efficacy [12], effective social support [13], and an ability to problem-solve [8].

Guatemala’s indigenous women manifest some of the worst health indicators worldwide [14]; three in four live below the poverty line [15]. Women of childbearing age living in indigenous areas show the highest rates of depression and anxiety in the country [16]. In rural indigenous Mam communities in the Western Highlands, lower household wealth, psychological distress, ineffective social support, inequality in decision-making, and experience of violence were consistent determinants of maternal stress, assessed via salivary cortisol, and infant stunting in the first 6 months of life [17]. Guatemala’s national health system provides limited access to mental health services; there are no formal mental health promotion and prevention programs, and limited involvement of service users and families in mental health systems [18, 19]. The Guatemalan civil war and long history of racial discrimination places indigenous populations at an additional disadvantage in terms of access to health services [20, 21].

Recent research has demonstrated the feasibility of psychosocial interventions for perinatal mental health in non-specialized health-care settings using psychoeducation [5, 22–24] cognitive restructuring [25, 26], problem-solving [25, 27, 28], behaviour activation [27], activating social networks [28, 29], and skilled parenting practices [30–32]. Few of these interventions have been tested in Latin America [27, 29] and none in indigenous populations. We addressed this knowledge gap for a population at special disadvantage of maternal mental health disorders through the co-design of a culturally safe perinatal group psychosocial intervention compatible with indigenous traditions – Women’s Circles.

The objective of this pilot randomised study was to assess co-designed Women’s Circles’ in terms of acceptability, feasibility and proof-of-concept in preparation for a future definitive trial.

## Methods

### Community involvement

Local women in the Mam communities (community health workers and traditional midwives, or *comadronas*) requested a group intervention – Women’s Circles – that could help and provide support for women in their communities, following earlier involvement in a participatory research project with the lead author of this paper [17]. In each community, local leaders steered group processes. We chose a participatory research approach [33, 34] to optimize community engagement and optimize cultural safety, acceptability and feasibility.

### Trial design

The design was a parallel group pilot randomised study.

### Setting

Five rural Mam communities in San Juan Ostuncalco municipality (25 km from Quetzaltenango city; population 1000-4000) and three periurban K’iche’ communities in Quetzaltenango city (population 4000-16,000) were selected as study sites, based on prior collaborations with the first author and local women leaders’ expressed interest in participating.

### Co-design

Ten six-hour workshops scheduled monthly with 16 circle leaders defined the transdiagnostic (addresses a range of mental health issues) intervention. Circle leaders collectively chose a project name and logo; developed a theory of change; mapped community needs, resources, and stakeholders; and pilot tested group methodologies. Group activities drew on games (*dinámicas*), art-based methods (drawing, role play, music) and group psychosocial therapy (active listening, emotion management, breathing and relaxation exercises, problem solving, popular education) to build trust, self-esteem, and social cohesion. Women’s interest in developing livelihood-sustaining skills prompted us to also incorporate productive activities (i.e. doll-making, crochet, cooking) as vocational therapy and potential income generation.

### Intervention

Additional file 1: Table S1 outlines the contents of the 10 sessions that followed a standard format. Pre-sessions involved toy-making of dolls, books or rattles mothers could use to stimulate and play with their infants. Sessions started with an inclusive participant-led prayer, followed by a prior session recap. A group game or *dinámica* served as an icebreaker. Activities that enabled personal and group reflection (drawing, dramatization) led to sharing lessons-learned, aspirations and personal experiences. A closing *dinámica* released tensions or promoted relaxation, through guided meditation or deep breathing exercises. Sessions concluded with a collective embrace. Sessions took place every fortnight in settings of participant’s choosing (i.e. house, community center), and lasted on average 2 h.

The intervention extended over 5 months, with sessions taking place every other week.

### Control

Control women did not receive an intervention but were invited to join a Women’s Circle when the post-intervention assessment was complete.

### Circle leaders

The 16 circle leaders were identified based on prior collaborations and expressed interest and invited to co-design and co-facilitate the intervention. Nine were former community health workers (CHWs), six were *comadronas* and one a community leader (former mayor). Aged 27 to 70 (mean 47.4 ± 14.5) years, one had no formal schooling, six had incomplete and five completed primary schooling and four had incomplete secondary schooling. Training by our research team lasted 50 h. After their own researcher-led 10-session Women’s Circle, where the 16 leaders acted as participants, they practiced session delivery (2/week, over 5 weeks). Additional training included crisis response, counselling, group facilitation and self-care skill-building. They received per diems of 50 quetzals (seven USD per day). All training activities were carried out in the leaders’ homes, on a rotating basis, as per their preference.

### Intervention fidelity

In the week preceding sessions, circle leaders joined a practice round. Ongoing support included phone debriefing and direct observation of a random sample of sessions, carried out with all leaders by our research team. The research team and more experienced circle leaders accompanied others facing difficulties.

### Participant selection

A checklist for participant eligibility included being pregnant or under 2 years postpartum and having at least one of the following conditions: socioeconomic disadvantage, domestic violence, difficult interpersonal relationships, poor social support, or psychological distress. These criteria were based on known risk factors [10], circle leaders’ assessment of what constituted maternal vulnerability, and prior research in nearby Mam communities [17]. Circle leaders visited eligible participants, explained the intervention and invited their participation.

We originally intended to recruit women who scored high on an initial screening test for symptoms of depression and anxiety; however, the absence of primary health care services in the target communities made it difficult to screen this population. Instead, the leaders thought it preferable to select participants based on known need and their own familiarity with local women. This method seemed realistic and feasible for future implementation of the study.

### Surveys

All participants underwent baseline and follow-up surveys. At enrolment, eligible women gave informed consent, a trained female interviewer (fluent in Spanish and Mam in Mam communities) administered the questionnaire, and nutritionists measured height and weight of her youngest child. A follow-up home-based assessment used the same questionnaire 1-month post-intervention. All instruments underwent pilot testing and semantic validation in Spanish. As few could read Mam or K’iche’, no Maya translations were performed; instead, data collectors agreed on vocabulary to be used with non-Spanish speakers. Surveys took between 20 and 30 min to complete.

### In-depth interviews

Post-intervention, a trained, bilingual Mam-Spanish female interviewer conducted in-depth interviews of 14 circle leaders and of two women participants in each of the seven communities still participating in the intervention, after obtaining informed consent. The script-based interviews lasted between 20 and 45 min, were conducted in a location of the women’s choice, and were audio-recorded.

### Outcomes

#### Primary outcomes

- Maternal symptoms of depression and anxiety over the last month, using the Hopkins Symptom Checklist-25 [35] (HSCL-25), a symptom inventory composed of a 10-item anxiety cluster, a 13-item depression cluster, and two additional somatic symptoms. Each item scores on a scale from one (not at all) to 4 (extremely); item scores can be summed to provide an estimate of the severity of anxiety and depression symptomatologies. A higher score indicates greater distress.
- Maternal wellbeing, using the Mental Health Continuum Short Form [36] (MHC-SF), comprised of 14 items representing the three dimensions of wellbeing: emotional, social and psychological. Each item scores on a scale from zero (never) to four (always), based on experiences in the previous month, allowing for continuous assessment of positive mental health. A higher score indicates greater wellbeing.

#### Secondary outcomes

- Self-efficacy measurement used a four-item subscale measuring self-efficacy in childcare (feeding, caring and cleaning, playing and talking, helping recover from illness) and a four-item subscale measuring self-efficacy in self-care (overcoming daily problems; staying calm when worried, nervous, or afraid; finding reliable people for support; dedicating time to herself). Each item scores on a scale from zero (I can’t do it) to three (I can do it), allowing for continuous assessment of childcare self-efficacy, self-care self-efficacy, and total self-efficacy. A higher score indicates greater self-efficacy.
- Mother’s engagement in early infant stimulation, using six items from the UNICEF Multiple Indicator Cluster Survey Early Child Development module capturing adult-child interactions [37], assessing whether mothers engaged with her infant in six different activities (e.g., reading, singing, playing, talking) over the preceding 3 days. Each item scored as zero (no) or one (yes). The cumulative number of activities was used as a continuous variable for analyses. Only women who had a child under 2 years old participated in this questionnaire. A higher score indicates greater involvement in early infant stimulation activities.

##### Sociodemographic information

Included: date of birth, age, ethnicity (Mam, K’iche’, non-indigenous), language proficiency (Mam, K’iche’, Spanish), marital status (married, informal-union, single, separated, widowed), occupation, formal schooling (none, primary, secondary, higher), parity and access to health providers (CHWs, doctors/nurses, traditional healers, religious leaders), relatives they lived with, and ownership of: electricity, faucet, toilet, refrigerator, computer, mobile phone, television, motorcycle/bicycle, car/truck or separate room for children.

##### Measures of acceptability

Acceptability was assessed in post-intervention in-depth interviews when participants were asked whether they were satisfied with the intervention, would recommend it to other women, and would have preferred it to be any different. We also asked participants to report on barriers to participation; circle leaders were asked what strategies they used to overcome these.

##### Measures of feasibility

Feasibility was assessed during post-intervention in-depth interviews. Circle leaders were asked whether they felt comfortable in their ability to lead the Women Circles, had received enough training and support, felt that implementation logistics were appropriate (i.e. session frequency, location and length, materials, compensation), and what they might change. Objective data included: rate of circle leader retention, rate of women participant retention, and number of sessions attended.

### Randomisation

#### Sequence generation and implementation of randomization

We used a non-computerised randomisation process. For each participating community, names of consenting women were put in a box and 12 names were drawn randomly to join the intervention group. Remaining names were allocated to the control group with a delayed circle intervention.

#### Allocation concealment and masking

In a study of this nature it is virtually impossible to keep allocation to groups concealed after the intervention starts. However, we made no announcements as to the allocation to any of the participants. Given the nature of the intervention, masking was not possible either.

#### Sample size

A total of 176 women in the eight participating communities met eligibility criteria. One community and its 16 participants withdrew from the study prior to randomisation. Without subsampling, in the remaining seven communities, we allocated 84 women to the intervention arm (one Women Circle of 12 women per community) and the remaining 71 were allocated to a control arm (with women spread over the seven communities).

### Data analysis

#### Data entry and security

Manual, double data entry of questionnaires minimized errors. Quantitative analyses relied on SPSS Statistics Program (version 22.0) for all primary and secondary analyses, and on CIETmap open-source software for supplementary analyses; all followed the intention to treat principle. In-depth interviews were transcribed verbatim in Spanish and analysed using MAXQDA 11 (version 11.2.1).

#### Baseline analysis

Descriptive statistics of demographic and psychosocial (outcome) measures were compared across groups (intervention vs control). All comparisons are accompanied by their corresponding *p*-values.

#### Primary analyses

We used independent sample t-tests comparing intervention vs control on the four mean psychosocial scores and their sub-scores to test for the potential effect of the intervention compared to the control group. All comparisons are accompanied by their corresponding *p*-values.

#### Secondary analyses

We compared outcome variables across groups using multiple linear regression analyses on each of the four outcome variables (a) prior to adjusting for other variables, (b) after adjusting for baseline values and (c) adjusting for baseline values, area of residence (rural vs. periurban), and maternal age; the latter data is shown. We also explored for a potential “dose response” (association between total number of sessions attended and outcome variables) among intervention women, using number of sessions attended as a continuous variable (zero to ten), performing the same analyses as described above. For all multiple regressions, we tested the following assumptions: Absence of multicollinearity (variance inflation factor, VIF < 2.5); Independence of residuals (Durbin Watson statistic between one and three); Variance of residuals, or homoscedasticity (scatterplot of residuals); and Normal distribution of residuals (normal P-P plot of residuals). Unstandardized coefficients (B) ± standard errors (SE) and *P*-value are reported.

#### Supplementary analyses

Given high baseline heterogeneity between rural and peri-urban areas, we used generalised estimating equation (GEE) for Logistic Regression in the R package Zelig [38] in an exchangeable correlation structure (logit.gee model, 1000 simulations, robust 95%CI) to evaluate for a potential cluster effect on psychosocial outcome variables. We created binomial scores for all primary and secondary outcome variables, using mean pre-intervention scores as cut-off (0 if ≤ mean, 1 if > mean). Analyses were adjusted for pre-intervention scores and maternal age. Odds ratios (OR), robust 95% confidence intervals (CI) and number needed to treat (NNT) are reported.

#### Missing data

We imputed missing data for individual items of the psychosocial questionnaires by calculating the mean of the remaining questionnaire items and replacing the missing data with that value. All other missing data (i.e. socio-demographics) were ignored.

#### Qualitative analyses

AB and PL independently analysed the data. MAXQDA 11 (version 11.2.1) was used to organize the data and code the transcripts via thematic content analysis [39]. Codes included dimensions of acceptability (i.e. affective attitude, burden, self-efficacy, perceived effectiveness, coherence), feasibility (i.e. barriers to participation) and dimensions of expected effectiveness (i.e. self-esteem, self-efficacy, social support, knowledge exchange, emotional wellbeing). AMC reviewed both analyses and extracted key dimensions and quotes for publication.

### Ethical review

Research ethics boards at the Douglas Mental Health University Institute (McGill University) and Institute of Nutrition of Central America and Panama (INCAP) in Guatemala approved the study. Community leaders and the Ministry of Health also granted permission. Each participant provided fully informed consent (signature or thumbprint) at enrolment. Circle leaders recruited and obtained informed consent of all participants.

## Results

**Fig. 1:**
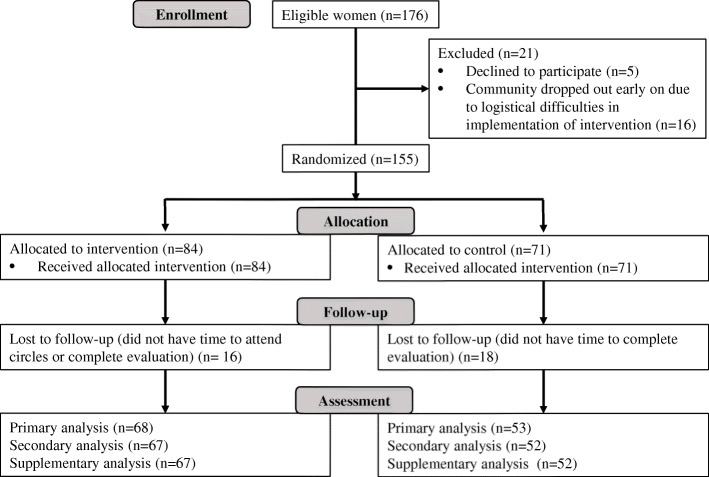
Participant flow diagram

Participant flow is shown in Fig. 1.

Fewer than 3% of eligible mothers did not provide consent. One periurban community and both its leaders and participants dropped out prior to randomisation due to local women’s time constraints related to employment, resulting in a final sample size of 155 women (84 intervention, 71 control) in seven communities, and 14 circle leaders.

### Recruitment

The pilot was successfully conducted within the stipulated period of 5 months in both settings. Recruitment strategies were successful; there were many women who met eligibility criteria and were interested in participating. No major untoward or unexpected incidents were reported.

### Numbers analysed

All recruited mothers were invited to complete the survey; out of the 155 study participants, 147 (81 intervention, 66 control) completed it at baseline, and 121 (68 intervention, 53 control) post-intervention.

The 84 intervention mothers attended a mean of 4.6 ± 3.6 sessions: 19% did not show up for any sessions, 17% attended one session, 10% attended two to four sessions and 55% five or more.

### Baseline data

Study participants were easily identified based on the defined eligibility criteria. The majority (73%) were selected for living in extreme poverty, 40% for experiencing psychosocial distress, and 3% for having family problems (categories are non-exclusive).

Mean maternal age was 26.2 ± 6.4 yrs. (15 to 43) (Table 1). Most (95%) rural mothers self-described as Mam, and 74% periurban mothers as K’iche’. The majority reported living in economically insecure households (57%); 59% had a stunted lastborn child, and 4% a wasted child.

**Table 1:** Baseline characteristics of participants in intervention vs. control groups: Mean, standard deviation and sample size (*N*) for continuous variables, and percent and sample size (*N*) for categorical variables

	Control	Intervention	*P*-value ^a^
Maternal age, yrs	26.2 ± 6.5 (65/66)	26.2 ± 6.3 (79/81)	0.953
Parity, #	2.6 ± 1.9 (62/66)	2.5 ± 1.6 (80/81)	0.802
Ethnicity, self-reported			
Not indigenous	7.3% (4/55)	12.7% (9/71)	0.327
*Mam*	78.2% (43/55)	66.2% (47/71)	
*K’iche’*	14.5% (8/55)	21.1% (15/71)	
Reproductive status			
Infant 0 to 2 years old	75.8% (50/66)	82.7% (67/81)	0.298
Pregnant	32.3% (21/65)	19.8% (16/81)	0.083
Marital status			
Married	47.0% (31/66)	39.5% (32/81)	0.218
Informal union, living with partner	47.0% (31/66)	45.7% (37/81)	
Single/widowed	6.1% (4/66)	14.8% (12/81)	
Formal schooling			
None	20.0% (11/55)	14.1% (10/71)	0.921
Incomplete primary	34.5% (19/55)	42.3% (30/71)	
Complete primary	23.6% (13/55)	21.1% (15/71)	
Incomplete secondary	14.5% (8/55)	11.3% (8/71)	
Complete secondary	5.5% (3/55)	9.9% (7/71)	
Higher education	1.8% (1/55)	1.4% (1/71)	
Profession			
Housewife	92.4% (61/66)	95.1% (77/81)	0.732
Living with...			
Mother	18.2% (12/66)	23.5% (19/81)	0.436
Mother-in-law	33.3% (22/66)	32.1% (26/81)	0.874
Partner	84.8% (56/66)	80.2% (65/81)	0.467
Economic security			
Economically insecure household	57.4% (31/54)	55.7% (39/70)	0.850
Household assets			
Electricity	7.3% (4/55)	14.1% (10/71)	0.228
Refrigerator	20.0% (11/55)	26.8% (19/71)	0.377
Computer	5.5% (3/55)	4.2% (3/71)	1.000
Cellphone	12.7% (7/55)	16.9% (12/71)	0.619
TV	58.2% (32/55)	57.7% (41/71)	0.961
Separate room for children	32.7% (18/55)	28.2% (20/71)	0.580
Motorcycle or bicycle	47.8% (11/55)	52.2% (12/71)	0.655
Car or truck	25.5% (14/55)	46.5% (33/71)	0.016
Toilet	96.4% (53/55)	98.6% (70/71)	0.580
Faucet	87.3% (48/55)	87.3% (62/71)	0.993
Access to physical/emotional health provider			
No one	18.4% (9/49)	34.8% (23/66)	0.051
Health worker	46.9% (23/49)	45.5% (30/66)	0.245
Doctor or nurse	63.3% (31/49)	45.5% (30/66)	0.425
Traditional healer	8.2% (4/49)	12.1% (8/66)	0.265
Religious leaders	20.4% (10/49)	19.7% (13/66)	0.595
Family	73.5% (36/49)	56.1% (37/66)	0.740
Infant nutritional status			
Stunting	61.2% (30/49)	56.7% (38/67)	0.349
Wasting	8.2% (4/49)	1.5% (1/67)	0.161

^a^*P*-value adjusted for multiple comparisons, significance level set at *p* < 0.001

There were no significant baseline differences between intervention and control women, in either sociodemographic (Table 1) or primary or secondary outcome (Table 2) measures.

**Table 2:** Baseline characteristics: T-test comparison of psychosocial scores of women participants, in intervention vs. control groups: Mean, standard deviation and sample size (*N*)

Psychosocial scores ^a^	Psychosocial scores		
	Control	Intervention	*p*-value
Psychosocial distress score (HSCL-25)			
Anxiety sub-score	15.4 ± 4.5 (66)	16.5 ± 5.0 (81)	0.144
Depression sub-score	20.1 ± 6.7 (66)	22.1 ± 6.9 (81)	0.083
Total score	35.5 ± 10.5 (66)	38.6 ± 11.3 (81)	0.088
Wellbeing score (MHC-SF)			
Total score	41.7 ± 12.8 (66)	42.6 ± 12.8 (81)	0.661
Self-efficacy score			
Infant care sub-score	11.0 ± 1.6 (49)	10.6 ± 2.1 (66)	0.271
Self-care sub-score	8.0 ± 2.3 (64)	8.0 ± 2.3 (80)	0.890
Total score	18.9 ± 3.2 (48)	18.4 ± 3.9 (66)	0.488
Infant stimulation engagement			
Total score	2.8 ± 3.6 (49)	2.7 ± 2.9 (66)	0.818

^a^A higher psychosocial distress score (HSCL-25) indicates greater distress; a higher wellbeing score (MHC-SF) indicates greater wellbeing; a higher auto-efficacy score indicates greater self-efficacy; a higher Infant stimulation engagement score indicates greater maternal engagement in infant stimulation activities

Since we found significant differences between rural and urban populations, we conducted additional analyses to understand this situation better. Rural compared to periurban women were more likely to have higher parity (2.8 ± 1.8 vs. 2.0 ± 1.2; *p*-value 0.008), be in an informal union (60 vs. 10%, *p*-value < 0.001) and have less schooling (20 vs. 7% never attended school, *p*-value < 0.001). They were less likely to be employed (1 vs. 23%, *p*-value < 0.001) and to own refrigerators, cell phones, televisions, car or trucks, or have a separate room for children (*p*-value < 0.05). Rural women had significantly lower anxiety sub-scores (15.5 ± 4.7 vs. 17.4 ± 4.8; *p*-value 0.045), total HSCL-25 scores (36.1 ± 11.4 vs. 40.1 ± 9.4; *p*-value 0.036), wellbeing scores (38.7 ± 12.3 vs. 51.5 ± 9.2; *p*-value < 0.001) and infant stimulation scores (1.7 ± 2.4 vs. 5.9 ± 3.5; *p*-value < 0.001).

### Outcomes and estimation

#### Primary analyses

Post-intervention, intervention compared to control women had a significantly higher MHC-SF score (greater wellbeing; 45.8 ± 10.5 vs. 40.2 ± 12.5; *p*-value 0.008) and a significantly higher self-care sub-score (greater self-efficacy in self-care; 9.2 ± 2.5 vs. 8.4 ± 2.0; *p*-value 0.049) (Table 3). However, there were no differences in the HSCL-25 scores or sub-scores, in the total self-efficacy score, or in the engagement in infant stimulation activities score.

**Table 3:** Primary analysis: T-test comparison of post-intervention psychosocial scores of women participants, in intervention vs. control groups: Mean, standard deviation and sample size (*N*)

Psychosocial scores ^a^	Post-intervention psychosocial scores		
	Control	Intervention	*p*-value
Psychosocial distress score (HSCL-25)			
Anxiety sub-score	15.3 ± 4.7 (53)	15.8 ± 4.4 (68)	0.561
Depression sub-score	20.4 ± 7.0 (53)	21.0 ± 6.6 (68)	0.658
Total score	35.7 ± 11.4 (53)	36.7 ± 10.7 (68)	0.608
Wellbeing score (MHC-SF)			
Total score	40.2 ± 12.5 (54)	45.8 ± 10.5 (68)	0.008
Self-efficacy score			
Infant care sub-score	10.9 ± 1.6 (38)	11.2 ± 1.7 (59)	0.446
Self-care sub-score	8.4 ± 2.0 (52)	9.2 ± 2.5 (68)	0.049
Total score	19.4 ± 3.2 (38)	20.5 ± 3.7 (59)	0.130
Infant stimulation engagement			
Total score	1.4 ± 2.0 (37)	1.9 ± 2.0 (59)	0.241

^a^A higher psychosocial distress score (HSCL-25) indicates greater distress; a higher wellbeing score (MHC-SF) indicates greater wellbeing; a higher auto-efficacy score indicates greater self-efficacy; a higher Infant stimulation engagement score indicates greater maternal engagement in infant stimulation activities

#### Secondary analyses

Multiple linear regression analysis revealed that intervention women were more likely to have a higher post-intervention MHC-SF score (greater wellbeing) than were control women (*p*-value 0.011) (Table 4). They were also significantly more likely to have a higher self-care sub-score score (greater self-efficacy in self-care) after controlling for baseline score only (data not shown; *p*-value 0.028); however, this difference became less apparent in the fully adjusted model (*p*-value 0.056). All assumptions are presented in Additional file 2: Table S2.

**Table 4:** Secondary analysis: Multiple linear regression models of study arm allocation (intervention vs. control) and psychosocial health scores, adjusted for maternal age, area of residence and baseline score: *B* = unstandardized coefficient, *SE* = standard error and sample size (*N*)

Psychosocial scores ^a^	*B* ± SE	*P*-value
Psychosocial distress score (HSCL-25) ^b^	−1.548 ± 1.418 (118)	0.277
Wellbeing score (MHC-SF)	4.707 ± 1.816 (119)	0.011
Self-efficacy score: Self-care sub-score	0.801 ± 0.415 (116)	0.056
Self-efficacy score: Infant care sub-score	−0.128 ± 0.380 (75)	0.737
Infant stimulation score	0.242 ± 0.395 (74)	0.611

^a^Increases in the HSCL-25, MHC-SF, self-efficacy and infant stimulation scores indicate greater distress, greater wellbeing, greater self-efficacy, and greater maternal engagement in infant stimulation activities, respectively. ^b^ Depression and anxiety sub-scores with similar findings, namely non-significant association with attendance; data not shown

Multiple linear regression analyses revealed several significant associations between number of sessions attended and primary and secondary outcome variables (Table 5). Having participated in a greater number of sessions was associated with having (1) a higher MHC-SF score (greater wellbeing; *p*-value 0.007), (2) a higher self-care sub-score (greater self-efficacy; *p*-value 0.014); (3) a higher infant-care sub-score (greater self-efficacy; *p*-value 0.043), and (4) a higher infant stimulation score (greater maternal participation in early infant stimulation; *p*-value 0.019). All assumptions are presented in Additional file 2: Table S2.

**Table 5:** Secondary analysis: Multiple linear regression models of number of sessions attended (0 to 10) and the psychosocial health scores, among mothers in the intervention arm, adjusted for maternal age, area of residence and baseline score: *B* = unstandardized coefficient, *SE* = standard error and sample size (*N*)

Psychosocial scores ^a^	*B* ± SE (N)	*P*-value
Psychosocial distress score (HSCL-25) ^b^	0.225 ± 0.242 (66)	0.358
Wellbeing score (MHC-SF)	0.819 ± 0.294 (66)	0.007
Self-efficacy score: Self-care sub-score	0.202 ± 0.080 (66)	0.014
Self-efficacy score: Infant care sub-score	0.141 ± 0.067 (47)	0.043
Infant stimulation score	0.165 ± 0.068 (46)	0.019

^a^Increases in the HSCL-25, MHC-SF, self-efficacy and infant stimulation scores indicate greater distress, greater wellbeing, greater self-efficacy, and greater maternal engagement in infant stimulation activities, respectively. ^b^ Depression and anxiety sub-scores with similar findings, namely non-significant association with attendance; data not shown

#### Supplementary analyses

GEE analyses revealed several significant associations between study arm allocation and primary and secondary outcome variables post-intervention, clustering by site (rural versus periurban) and adjusting for pre-intervention score and maternal age (Table 6). Relative to control, the intervention increased both the MCH-SF score (greater wellbeing, OR 2.01, 95% CI 1.39–2.89) and the self-care self-efficacy sub-score (greater self-efficacy, OR 2.02, 95% CI 1.22–3.35); and decreased the HSCL-25 score (lessened psychosocial distress, OR 0.86, 95% CI 0.85–0.86). Numbers needed to treat (NNT) were 6, 6 ad 33, respectively.

**Table 6:** Supplementary analysis: General Estimating Equation for Logistic Regression of study arm allocation (intervention vs. control) and psychosocial health scores, clustering for area of residence (rural vs. periurban) and adjusted for baseline score and maternal age: *OR* = odds ratio, *NNT* = number needed to treat, *CI* = confidence interval

Psychosocial scores ^a^	OR (95% CI)	NNT (95% CI)
Psychosocial distress score (HSCL-25) ^b^	0.86 (0.85–0.86)	33 (33–33)
Wellbeing score (MHC-SF)	2.01 (1.39–2.89)	6 (4–13)
Self-efficacy score: Self-care sub-score	2.02 (1.22–3.35)	6 (4–25)
Self-efficacy score: Infant care sub-score	1.55 (0.68–3.54)	13 (−11–5)
Self-efficacy score: Total	1.14 (0.84–1.56)	50 (−20–13)
Infant stimulation score	1.2 (0.90–1.60)	33 (− 50–17)

^a^Increases in the HSCL-25, MHC-SF, self-efficacy and infant stimulation scores indicate greater distress, greater wellbeing, greater self-efficacy, and greater maternal engagement in infant stimulation activities, respectively. ^b^ Analyses with HSCL-25 anxiety and depression sub-scores were not significant

### Acceptability and feasibility

#### Circle leaders as delivery-agents

All mothers felt comfortable with the circle leaders and that they could trust them.

A few circle leaders had initially been hesitant about their ability to lead a group intervention. Post-intervention, all expressed satisfaction from their role and saw it as a positive experience. They appreciated learning new knowledge and skills, helping other women, and making a meaningful contribution to their community. One young leader expressed, “I am happy, because now I am no longer afraid [of leading the sessions]. Initially I was very anxious, but after a while it became easier, and the women liked all the sessions, and some of them now come to me to talk about their problems”.

Most leaders reported having time to fulfill their role. Occasional scheduling difficulties were related to personal (i.e. religious) or work (i.e. agriculture, attending deliveries, other projects) obligations. The majority were satisfied with the training and supervision received, feeling it strengthened their knowledge and leadership skills and adequately prepared them. The manual was a useful reference. They reported initiating various engagement strategies, including: visiting mothers in their homes; arranging the timing, duration and location of the sessions at the mothers’ convenience; preparing food; and adapting and creating new activities to meet mothers’ needs and interests (including between sessions with productive activities).

#### Views about the intervention

All participants and leaders thought the intervention was a positive experience, and most requested it be continued. One woman shared, “We learned many new things from one another and we also had fun and laughed with other women and shared what we felt in our hearts. Coming to the Circles helped us forget our worries for a while and spend a pleasant moment.”

The majority of women appreciated the manner in which the sessions were held, including the play- and arts-based activities, which enabled them to “relax, release tensions and feel positive emotions”. Some themes (i.e. early infant stimulation) and activities (i.e. cooking, handicraft-making) were especially appreciated. Several reported sharing content with trusted family, most often that touching on self-esteem, childrearing, early infant stimulation and the family economy: “When [this participant] returns home, her mother-in-law asks her what she learned, and she shares what she learns with her, and she shares the exercises with her” [leader].

Only in one periurban community did women oppose the sessions on inter-partner violence and reproductive health.

The leaders reported that session delivery took longer than anticipated and suggested reducing content or splitting them into various sessions. Most mothers and leaders suggested holding more frequent productive workshops (i.e. every other week), alternating them with the more theoretical ones.

#### Effectiveness

##### Self-esteem and agency

Most participants and leaders felt the Circles had positively impacted their self-esteem. Many women said they learned to value themselves (“As women, we hardly ever value ourselves, here in our community”), as captured by one participant:

I used to place much importance on what others said about me, and this made me feel bad or sad. My mother-in-law often told me I was stupid, not worth anything. Now I take a bit of time every day to see myself and make myself feel better about who I am and what I know how to do. I now try not to place so much importance on what they say and excuse myself from people who are being offensive. I go for a walk when they start insulting me at home.

Another woman explained: “A woman is afraid of her husband, he is in charge. But I learned a woman also has the right to speak or express her opinions. This is what I am happiest about, because a woman may be pretty or ugly or whatever, she has the right to speak her mind too.” A leader described how one participant, who used to have low self-esteem, told her husband that she would decide what to do with her life, and not just follow his or his mother’s wishes: “She had the courage to speak up, and she decided where she would deliver her baby.”

One leader mentioned that her participation had “helped her become stronger, braver, and to not let myself be overcome by anything.” Another shared,

The activity I prefer is when women draw their personal map, in the shape of a tree. We are as plants. We have roots too. It is only that sometimes we don’t value who we are, what we hold in our arms, what we can do. This is what has most caught my attention. It doesn’t leave my mind.

##### Improved emotional health and wellbeing

Many mothers and leaders thought the sessions helped them gain perspective and agonize less over their worries. One woman shared: “I used to feel my head didn’t work, I was forgetting everything. When I started attending [the Circles], my heart stopped hurting from all the sadness and my head stopped hurting from thinking too much, and I could start thinking clearly again, and do what I needed to do.” One leader mentioned: “Many women leave their homes heavy with worries, sadness and problems. But with the activities they start laughing and feeling happy.” Another shared: “The sessions help them relax, and when they leave, they go with a smile. You can see the impact on their faces.”

##### Improved self-efficacy

Several women mentioned they used to spend a lot of time at home thinking about their problems without knowing what to do. Many found it useful to hear what others go through and share experiences and solutions: “It helps to get things off one’s chest, and speak about ones’ problems with others, to not make problems bigger than they are, and gain knowledge that can help solve them.” A young leader shared that her participation had “helped her plan her life and think about her dreams.”

##### Improved social support and relationships

Women and leaders mentioned that most women in the community have few people to turn to for support. Several women mentioned having no one, while others only had their husband or mother. Most thought the intervention allowed them to develop trusting relationships with other women and to feel listened to and newly supported. One woman commented, “It was especially nice sharing with other women. Often, we do not get along well with other women. But here, through the activities, you start getting along with other women and you get to know them. This doesn’t happen often here.”

A leader summarized, “The women realized that it was worth it to come to the sessions and share advice with others, and little by little more and more give advice to one another and share experiences and help one another.” Another mentioned, “The women accepted one another as sisters. There were some who were a bit proud at first, and others who were shy, but together they all pulled each other up.”

Several women mentioned that the Circles helped them improve their relationship with their children and other family members, because they learned to better manage their anger and to not make others feel bad by insulting or hitting them.

##### Barriers and potential untoward effects

Neither the mothers nor the leaders were aware of any untoward effects of participation.

Several women were initially anxious for not knowing other participants and worried about sensitive information being shared outside the group. Kinships within the same group (i.e. in-laws) sometimes limited sharing freely. Additionally, tensions existed between some participants, as one woman described: “Maybe she does not like me, because she sells pigs and doesn’t want me to sell pigs too.” Another expressed, “At first we felt embarrassed and fearful to speak. For example, one woman always tried to make us feel bad. She made fun of us when we could not do or say things well.” However, these fears and tensions tended to abate over time; “Little by little I started feeling more comfortable with the other women, we shared concerns and enjoyed being together.”

A few women felt intimidated by lack of Spanish fluency and a lower level of literacy. More literate women participated more actively. As mentioned by one leader, “at first they felt insecure or embarrassed, and said they couldn’t do things we asked them to do, but now they do them, and they laugh and have fun, as if they lost fear, and little by little realized they can do many things.”

Some women had to overcome family restrictions to participate, mostly from mothers-in-laws. A few participated secretly:

There are people who make fun of women for participating in such activities, who ask, “Why do you go there? Don’t you have any work? What good is it for you?” Here in the community people make fun of what you do all the time. And so, I am afraid. I won’t lie to you, I haven’t told anyone that I am participating.

Interestingly, most women were able to obtain their partners’ consent. One woman stated, “When I asked for my husband’s authorization, he asked me what I was going to do there, and I explained everything to him, and he told me that it was good, that I was going to be learning things, that they were very good things to be learning about.” Several husbands reminded the women to go the sessions and supported them, so they could leave home for a while.

Sharing the sessions’ content with husbands and mothers-in-law sometimes increased their support.

Inclement weather and harvesting presented occasional barriers to participation. Although poor attendance was usually explained by time constraints and women’s workload, not offering gifts or material goods made it harder to motivate mothers. Not having help with childcare was also a barrier.

## Discussion

### Principal findings

The intervention was feasible, acceptable and potentially efficacious in that it was reported as providing a unique environment for leaders and women participants to learn, increase their self-esteem, improve their emotional wellbeing, discuss and solve their problems, and gain new sources of support and friendship. The intervention seems to have also increased maternal wellbeing, self-efficacy and engagement in early infant stimulation activities; it also had a clustered reduction on psychosocial distress.

### Community participation

This pilot demonstrated acceptability and feasibility of intervention co-design by local women, in a historically excluded population rarely consulted in decision-making processes relating to their own health. The fields of social community psychology, critical psychology and liberation psychology have long argued for psychosocial interventions that contribute to strengthening people’s possibilities for solving their own problems (conscientization, problematization) in contexts of oppression, struggle and post-colonialism [40, 41] and allow them to become active agents in their own transformation (social mobilization) [42]. This is of particular relevance to Guatemala’s indigenous populations, where most psychosocial problems can be traced to the daily stresses of poverty, discrimination, structural violence and a weakened post-conflict social fabric [43, 44]. The circle leaders assumed a role as catalysts of change. Interventions that address psychosocial determinants of health and wellbeing (i.e. lack of social support, or poor self-esteem, self-efficacy and problem-solving skills) are likely to have a long-term impact on the prevalence of perinatal common mental health disorders and on maternal and child health [45, 46].

### Lay health workers as circle leaders

Our findings add to the accruing evidence from LMIC that non-mental health specialists such as CHWs [30–32] and local women peers [22, 28, 47, 48] can be effective delivery-agents of psychosocial interventions, including group interventions [28, 48–50]. This has important implications in yet another context where health professionals are scarce [15] and where populations are additionally weary of consulting formal health services [20]. As in other studies [22, 26, 31], our leaders received focused training and ongoing supervision. They shared mothers’ sociocultural context and already held their community’s trust and support, allowing them to access mothers and take on their new role with relative ease and increasing the intervention’s cultural safety and acceptability. The impact that participating in the intervention (first as participants and then as leaders) had on the circle leaders’ own wellbeing validates using a cascade approach for its delivery and speaks to the need for also addressing community-based health professionals’ psychosocial health needs.

To the best of our knowledge, this is the first report of paired group leadership of a group psychosocial intervention aiming to represent both formal and informal health systems, and of traditional midwives (*comadronas*) acting as delivery-agents. The systemic neglect of the role of traditional culture in health has been described as the single biggest barrier to advancement of the highest attainable standard of health worldwide, especially among marginalized groups [51]. In Guatemala, relations between formal and traditional providers are often tense due to differing approaches to health, a long history of discrimination and devaluation of indigenous knowledge and practices [20]. The overwhelming recourse to *comadronas* by indigenous women testifies to local cultural norms and preferences and greater trust in traditional practices [52–54]. C*omadronas’* unique contributions to women’s psychosocial health would be worth elucidating further, as would be their ability to transmit resilience factors and endogenous resources rooted in the local context.

### Multi-modal collective approach

Our pilot study suggests that a multi-modal approach is acceptable, feasible and effective. The small number of psychological interventions in LMICs – with none including indigenous or other marginalized populations of Latin America – limits their generalizability to our population. A meta-analysis combining trials from high-income countries (UK, Australia, Canada, USA, Germany) and two LMICs (India [28], China [55]) suggests that individual, multi-contact, and interpersonal therapy-based interventions may be most effective in preventing postnatal depression [56]. A recent meta-analysis of psychological interventions delivered by non-specialist mental health care providers in LMICs found a pooled reduction in maternal depression, but the heterogeneity of approaches did not permit comparisons between modalities [5]. Interventions based on biomedical models of mental illness have proven insufficient for addressing the needs of indigenous communities [57, 58], and there have been calls instead for a collective, holistic, strengths-based approaches rooted in cultural identity [59–61].

Our own approach allowed local women to find in the Women’s Circles a space that responded to their individual needs and interests, within a mutually supportive environment. A group rather than individual-focused intervention emerged as the delivery method of choice in our setting, contributing to the accruing evidence from LMICs that group interventions can be effective in improving maternal mental health [23]. Popular education and arts-based methods emerged as particularly powerful tools to facilitate women’s engagement with the stresses present in their daily lives and explorations of a better future.

### Strengths and limitations

The screening tool may not have detected all truly eligible women and included only those available to join the Circles. We may have excluded working mothers, women facing particularly harsh living conditions, women not given permission to participate, or women with poor levels of trust. Session attendance was not ideal; better selection of women based on interest and need may help increase retention, as might be adding in more productive activities, as suggested by participants. Local acceptability of the intervention was likely influenced by human resource elements that may be hard to replicate, built by project lead over the course of many years.

The pilot trial was not powered to test statistically significant differences in outcome variables, but rather the intervention’s feasibility and acceptability – making us wary of over-interpreting the measured impact. We recognize the limits of our statistical approach (i.e. small power, lack of clinical corollary, assumptions of linear regression), however the consistency of findings across methods used (primary, secondary and supplementary analyses) as well as with qualitative finding strengthens their credibility.

The HSCL-25 instrument may have lacked sensitivity in our patient population, something that we will need to investigate further. We do not provide comparisons with other studies because we consider that sociocultural contexts would be so different that it makes this exercise redundant and impossible to reach conclusions. The main dynamic behind impact may have been women’s empowerment – a challenging construct to quantify. We considered the possibility of positive sociability [22], with participants reporting more positive outcomes so that the project might continue. Circle leaders’ own aspirations for continued employment may have resulted in them painting a more positive picture of their experiences.

Although the use of non-mental health specialists is a potentially low-cost strategy to increase women’s access to evidence-based psychosocial care, its sustainability and scalability will need to be further explored [62]. Strategic nesting of the intervention into existing community-based maternal health programs [63] and relying on CHWs as delivery agents could reduce costs and ease referral to specialist care [27, 64], but also run the risk of overburdening fragile health systems, especially as psychosocial interventions are human-resource intensive. Intervention co-design may be challenging to reproduce in an institutional setting. Mechanisms to effectively support circle leaders to deliver the intervention within their communities need to be further explored. Women’s interest in having more productive activities included in the intervention could be explored as a self-sustaining income-generating mechanism.

Finally, the pilot was conducted within a specific context and we need to use caution in generalizing findings to other settings. The intervention will need to be adapted to the diverse contexts of Guatemala to enable scaling-up. It would also benefit from complementary *enabling* strategies; psychological interventions alone may not be sufficient when major contributing factors to women’s psychosocial distress are systemic and structural [10]. Where strong gender inequalities exist, it may be unrealistic to expect an intervention to empower women in a way that they are individually able to negotiate for a change in their lives [23]; involving men and communities is critical.

## Conclusions

To the best of our knowledge, this is the first psychosocial intervention that engaged end-users as partners in program co-design, helping to guarantee cultural safety and acceptability. An important innovation in mental health, the approach has special relevance in settings without formal mental health services. The Women’s Circle intervention emerges as a promising strategy in end-user engagement and community-based mental health promotion and prevention,

This study illustrates the feasibility of a holistic, community-based, peer-led psychosocial intervention for indigenous women in Latin America. Research findings with Maya mothers in Guatemala suggest that women’s groups can be leveraged as a critical space where mothers can engage in concrete actions to transform their lives. If, as postulated, high levels of psychosocial adversity affect not only a mother’s wellbeing but also her infant’s growth, development and life opportunity, increasing her ability to overcome adversity and psychosocial distress opens new possibilities for breaking vicious cycles of poverty, illness, and mental distress that plague marginalized communities.

## Additional files

Additional file 1: **Table S1.** Themes and content overview of Women’s Circles. This table outlines the contents (themes and objectives) of the 10 Women’s Circle sessions. (DOCX 17 kb)

Additional file 2: **Table S2.** Linear Regression Assumptions: Absence of multicollinearity (variance inflation factor, or VIF < 2.5), Independence of residuals (Durbin-Watson statistic between 1 and 3), Variance of residuals, or homoscedasticity (scatterplot of residuals), and Normal distribution of residuals (normal P-P plot of residuals). A. Linear regression assumptions for Table 4: Multiple linear regression models, adjusted for maternal age, area of residence and baseline score. B. Linear regression assumptions for Table 5: Multiple linear regression models, adjusted for maternal age, area of residence and baseline score. This table presents all assumptions that were tested prior to carrying out the multiple linear regression analyses, presented in the manuscript’s Tables 4 and 5. These include, as described in the manuscript under Analyses and in the Additional file Table: Absence of multicollinearity (variance inflation factor, or VIF < 2.5), Independence of residuals (Durbin-Watson statistic between 1 and 3), Variance of residuals, or homoscedasticity (scatterplot of residuals), and Normal distribution of residuals (normal P-P plot of residuals). (DOCX 27 kb)

## Abbreviations

CBT: Cognitive behavioural therapy
CHW: Community health workers
HSCL-25: Hopkins Symptom Checklist-25
INCAP: Institute of Nutrition of Central America and Panama
IPT: Interpersonal therapy
LMIC: Low- and middle-income countries
MHC-SF: Mental Health Continuum Short Form
UK: United Kingdom
UNICEF: United Nations Children’s Fund
USA: United States of America
