# Supplementary material for: Women’s circles as a culturally safe psychosocial intervention in Guatemalan indigenous communities: a community-led pilot randomised trial
Source: BMC Womens Health. 2019 Apr 3;19:53. doi: 10.1186/s12905-019-0744-z (PMC6448212; doi:10.1186/s12905-019-0744-z)
Supplement: Supplementary file 1 — Table S1. Themes and content overview of Women’s Circles. This table outlines the contents (themes and objectives) of the 10 Women’s Circle sessions. (DOCX 17 kb) [file 12905_2019_744_MOESM1_ESM.docx]

Additional file 1 Table S1. Themes and content overview of Women´s Circles.

| **Session** | **Theme** | **Objectives** |
| --- | --- | --- |
| 1 | Introduction: Getting to know each other and reflecting on the importance of creating Women´s Circles | Generate the first interactions among participants and promote their interest both through individual and collective reflections |
| 2 | Exploring my personal path within the Women´s Circle: Who am I? Who do I wish to be? | Promote critical participation through personal reflection on the life difficulties that interfere in achieving goals and promote the identification of the first step to overcoming these. |
| 3 | My priorities as a woman, as a mother | Create a space of reflection on the importance of health and wellbeing of women and their children |
| 4 | I am a woman, I am a mother: my nutrition | Promote critical thinking about the importance of women’s nutrition. Create opportunities for sharing experiences on women´s health and nutrition |
| 5 | My health as a woman: How can I protect myself from violence and its impacts on my health? | Promote critical reflection through individual and group reflection on violence and how it affects the health of women and their families, exploring strategies to tackle it. |
| 6 | Building my path as a woman, as a mother | Foster an environment for reflection and self-care of participants’ emotional state as women and mothers. Create opportunities for reflection, analysis, relaxation and healing. |
| 7 | My role in my household’s economy | Foster an environment for reflection on women’s contributions to the household economy. |
| 8 | My health as a woman and caring for my body: thinking about my reproductive health | Foster an environment for reflection and self-care of women’s reproductive and sexual health and its importance for the overall wellbeing of women |
| 9 | Early infant stimulation: Why it is important and what I can do? | Promote critical reflection on the importance of motor, physical and mental development of infants. Share knowledge and techniques to engage with and stimulate infants of different ages. |
| 10 | Closure: Helping women to help themselves, towards the sustainability of Women´s Circles | Prompt reflection on the contribution of their participation in the Women’s Circles on their health and wellbeing. Create viable and pertinent sustainability mechanisms for the operation and continuity of Women´s Circle activities. |
