# Supplementary material for: Women’s circles as a culturally safe psychosocial intervention in Guatemalan indigenous communities: a community-led pilot randomised trial
Source: BMC Womens Health. 2019 Apr 3;19:53. doi: 10.1186/s12905-019-0744-z (PMC6448212; doi:10.1186/s12905-019-0744-z)
Supplement: Supplementary file 2 — Table S2. Linear Regression Assumptions: Absence of multicollinearity (variance inflation factor, or VIF < 2.5), Independence of residuals (Durbin-Watson statistic between 1 and 3), Variance of residuals, or homoscedasticity (scatterplot of residuals), and Normal distribution of residuals (normal P-P plot of residuals). A. Linear regression assumptions for Table 4: Multiple linear regression models, adjusted for maternal age, area of residence and baseline score. B. Linear regression assumptions for Table 5: Multiple linear regression models, adjusted for maternal age, area of residence and baseline score. This table presents all assumptions that were tested prior to carrying out the multiple linear regression analyses, presented in the manuscript’s Tables 4 and 5. These include, as described in the manuscript under Analyses and in the Additional file Table: Absence of multicollinearity (variance inflation factor, or VIF < 2.5), Independence of residuals (Durbin-Watson statistic between 1 and 3), Variance of residuals, or homoscedasticity (scatterplot of residuals), and Normal distribution of residuals (normal P-P plot of residuals). (DOCX 27 kb) [file 12905_2019_744_MOESM2_ESM.docx]

Additional file 2 Table S2. Linear Regression Assumptions: Absence of multicollinearity (variance inflation factor, or VIF < 2.5), Independence of residuals (Durbin-Watson statistic between 1 and 3), Variance of residuals, or homoscedasticity (scatterplot of residuals), and Normal distribution of residuals (normal P-P plot of residuals)

1. Additional file 2 Table S2A. Linear regression assumptions for Table 4: Multiple linear regression models, adjusted for maternal age, area of residence and baseline score

| Multiple linear regression models | Absence of multicollinearity (VIF) | Independence of residuals (Durbin Watson statistic) | Variance of residuals (homoscedasticity) | Normal distribution of residuals |
| --- | --- | --- | --- | --- |
| Psychosocial distress score (HSCL-25) | 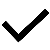 | 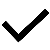 | 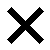 | 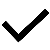 |
| Wellbeing score (MHC-SF) | 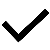 | 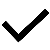 | 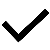 | 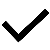 |
| Self-efficacy score: Self-care sub-score | 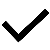 | 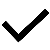 | 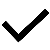 | 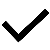 |
| Self-efficacy score: Infant care sub-score | 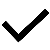 | 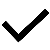 | 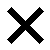 | 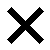 |
| Infant stimulation score | 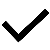 | 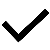 | 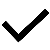 | 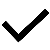 |

1. Additional file 2 Table S2B. Linear regression assumptions for Table 5: Multiple linear regression models, adjusted for maternal age, area of residence and baseline score

| Multiple linear regression models | Absence of multicollinearity (VIF) | Independence of residuals (Durbin Watson statistic) | Variance of residuals (homoscedasticity) | Normal distribution of residuals |
| --- | --- | --- | --- | --- |
| Psychosocial distress score (HSCL-25) | 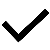 | 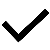 | 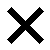 | 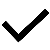 |
| Wellbeing score (MHC-SF) | 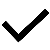 | 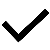 | 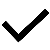 | 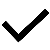 |
| Self-efficacy score: Self-care sub-score | 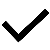 | 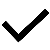 | 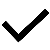 | 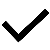 |
| Self-efficacy score: Infant care sub-score | 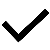 | 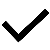 | 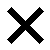 | 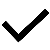 |
| Infant stimulation score | 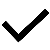 | 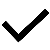 | 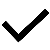 | 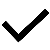 |
